# Trimetazidine in Heart Failure: From Metabolic Modulation to Clinical Outcomes—A Narrative Review

**DOI:** 10.3390/ph19101663

**Published:** 2026-10-21

**Authors:** Akshyaya Pradhan, Sudesh Prajapathi, Kunal Mahajan, Akash Batta, Marco Alfonso Perrone

**Affiliations:** 1 Department of Cardiology, King George’s Medical University, Lucknow 226003, Uttar Pradesh, India; akshyaya33@gmail.com; 2 Department of Cardiology, All India Institute of Medical Sciences, Bhopal 462020, Madhya Pradesh, India; sudeshprajapathi@gmail.com; 3 Himachal Heart Institute, Mandi 175021, Himachal Pradesh, India; kunalmahajan442@gmail.com; 4 Department of Cardiology, Dayanand Medical College and Hospital (DMCH), Ludhiana 141001, Punjab, India; akashbatta02@gmail.com; 5 Division of Cardiology and CardioLab, Department of Clinical Sciences and Translational Medicine, University of Rome Tor Vergata, 00133 Rome, Italy

**Keywords:** heart failure, trimetazidine, myocardial energy metabolism, metabolic therapy, heart failure with reduced ejection fraction, ventricular remodeling, oxidative stress, cardioprotection, glucose oxidation, mitochondrial dysfunction

## Abstract

Heart Failure (HF) remains a leading cause of morbidity and mortality worldwide despite major advances in guideline-directed medical therapy. Metabolic remodeling with impaired myocardial energy utilization has emerged as an important therapeutic target in HF. Trimetazidine, a metabolic modulator that partially inhibits fatty acid beta-oxidation and favours glucose oxidation, enhances myocardial energy efficiency without changing systemic hemodynamics. This narrative review summarizes the molecular mechanisms underlying the cardioprotective effects of trimetazidine and critically evaluates the available clinical evidence supporting its use in HF. Experimental studies have shown that trimetazidine increases ATP production, maintains mitochondrial function, decreases oxidative stress, inhibits apoptosis of cardiomyocytes, regulates autophagy, and reduces myocardial inflammation and fibrosis. Clinical studies and meta-analyses have shown that adjunctive therapy with trimetazidine has been associated with improved left ventricular ejection fraction, ventricular remodeling, exercise capacity, New York Heart Association functional class, biomarkers of myocardial injury, and quality of life in patients with heart failure with reduced ejection fraction. The available evidence also indicates reductions in cardiovascular hospitalization and possibly all-cause mortality, but these findings are based primarily on relatively small randomized trials and pooled analyses, and need confirmation in adequately powered multicenter studies. The potential role of trimetazidine alongside contemporary therapies, including sodium-glucose cotransporter-2 inhibitors and other guideline-directed treatments, is also discussed. Overall, trimetazidine appears to be a promising adjunctive metabolic therapy that may complement existing pharmacological strategies in selected patients with heart failure (Central Illustration). Further large-scale clinical trials are needed to better define its place in contemporary HF management.

## 1. Introduction

Heart failure signifies a key and mounting public health issue. Its estimated overall prevalence is 0.3–2%, which increases to more than 10% in the elderly population [1]. The rate of heart failure almost doubles with every decade of life [2]. Consequently, hospitalization rates have also shown a steady rise in the elderly. Heart failure is associated with an average one-year mortality of 33% and 5-year mortality of 60% [1,2]. Thus, in spite of a plethora of pharmacological agents available for the management of heart failure, the prognosis and mortality remain high.

Though our understanding of heart failure has enhanced immensely, we have limited pharmacotherapeutic options that act via neurohormonal inhibition, the adrenergic system, and regulating myocardial oxygen use via ventricular unloading [3]. Nevertheless, there is a need to focus on agents directed towards regulation of cardiac metabolism given the vital role of metabolic remodeling in heart failure, and the fact that the heart is an organ with a high metabolic rate [4].

## 2. Literature Search Strategy

PubMed/MEDLINE, Embase, and the Cochrane Library were searched from inception to [July 2026] using combinations of the terms “trimetazidine,” “heart failure,” “cardiomyopathy,” “left ventricular dysfunction,” “myocardial metabolism,” “fatty acid oxidation,” and “metabolic modulation.” Reference lists of retrieved reviews and meta-analyses were hand-searched. English-language randomized controlled trials, meta-analyses, mechanistic and experimental studies, and current guideline documents were prioritised; isolated case reports and conference abstracts without subsequent full publication were not considered. Source selection reflected the authors’ judgement of relevance and methodological quality, consistent with a narrative review; no formal risk-of-bias assessment or quantitative pooling was undertaken.

## 3. Cardiac Energetics in HF

The healthy heart incessantly produces large amounts of adenosine triphosphate (ATP) to maintain contractile function. The predominant process of ATP production in the heart is aerobic oxidation. In heart failure, the energy supply is deficient, which leads to the heart favouring glucose, resulting in enhanced oxidation efficiency. Thus, there is increased aerobic oxidation of fatty acids and decreased utilization of glucose [5]. FFA oxidation produces more ATP compared to glycolysis, but its energy efficiency is poorer due to excess consumption of oxygen. Additionally, glycolysis is enhanced, resulting in elevated levels of hydrogen ions and lactic acid within the myocardium, inhibiting the contractile function. Thus, in a patient with heart failure, due to considerable alterations in energy output, failure of contractile myocardial fibers occurs [3,6]. In such cases, a primary protective mechanism could involve attenuating FFA oxidation and enhanced glucose utilization. This is an adaptive mechanism observed in a failing heart. Therefore, an agent that could switch the metabolism from FFA to glucose could be therapeutic.

## 4. Mechanism of Trimetazidine

Trimetazidine reduces the rate of aerobic oxidation of fatty acids [7,8]. Therefore, it enhances the productivity of aerobic oxidation and also alleviates the production of hydrogen ions by glycolysis. It also encourages the production of mir-21, which suppresses PTEN activity, and consequently initiates the PI3K-Akt signal pathway, ultimately inhibiting the cardiomyocyte apoptosis pathway [9]. It also supports the expression of NADPH oxidase 2 and diminishes ROS. It modulates the cardiomyocyte autophagy in a bidirectional manner by regulating AMPK and Akt/mTOR [5]. Trimetazidine causes a higher turnover and accumulation of long-chain fatty acids into phospholipids, decreasing the fraction of phospholipids containing linoleic acid and increasing the content of phospholipids of cellular membranes [4,5].

Trimetazidine’s cardioprotective effect is mainly due to optimization of myocardial energy metabolism. Specifically, it inhibits mitochondrial long-chain 3-ketoacyl coenzyme A thiolase (3-KAT), a key enzyme in the final step of fatty acid β-oxidation [6,8]. Partial inhibition shifts myocardial substrate utilization from fatty acid oxidation toward glucose oxidation, which produces more ATP per molecule of oxygen consumed. Thus, myocardial oxygen utilization is improved, an effect that may be especially advantageous in the failing heart where oxygen delivery is compromised. Trimetazidine improves the myocardial contractile efficiency and protects the cellular energy reserves by restoring the balance between glycolysis and glucose oxidation, which leads to a decrease of intracellular acidosis and lactate accumulation.

Trimetazidine possesses several pleiotropic effects that are beneficial in myocardial protection apart from its metabolic actions. Experimental studies have also shown that it maintains mitochondrial integrity, inhibits the formation of reactive oxygen species and decreases oxidative stress through modulation of NADPH oxidase activity and enhancement of endogenous antioxidant defenses [5,7,9]. These actions reduce lipid peroxidation and limit oxidative damage to the cardiomyocytes. Moreover, trimetazidine activates prosurvival signalling pathways including the PI3K/Akt pathway via miR-21-mediated inhibition of PTEN, thereby inhibiting apoptosis [5]. It modulates autophagy through AMPK and Akt/mTOR signalling pathways to maintain cellular homeostasis during metabolic stress.

**Figure 1:**
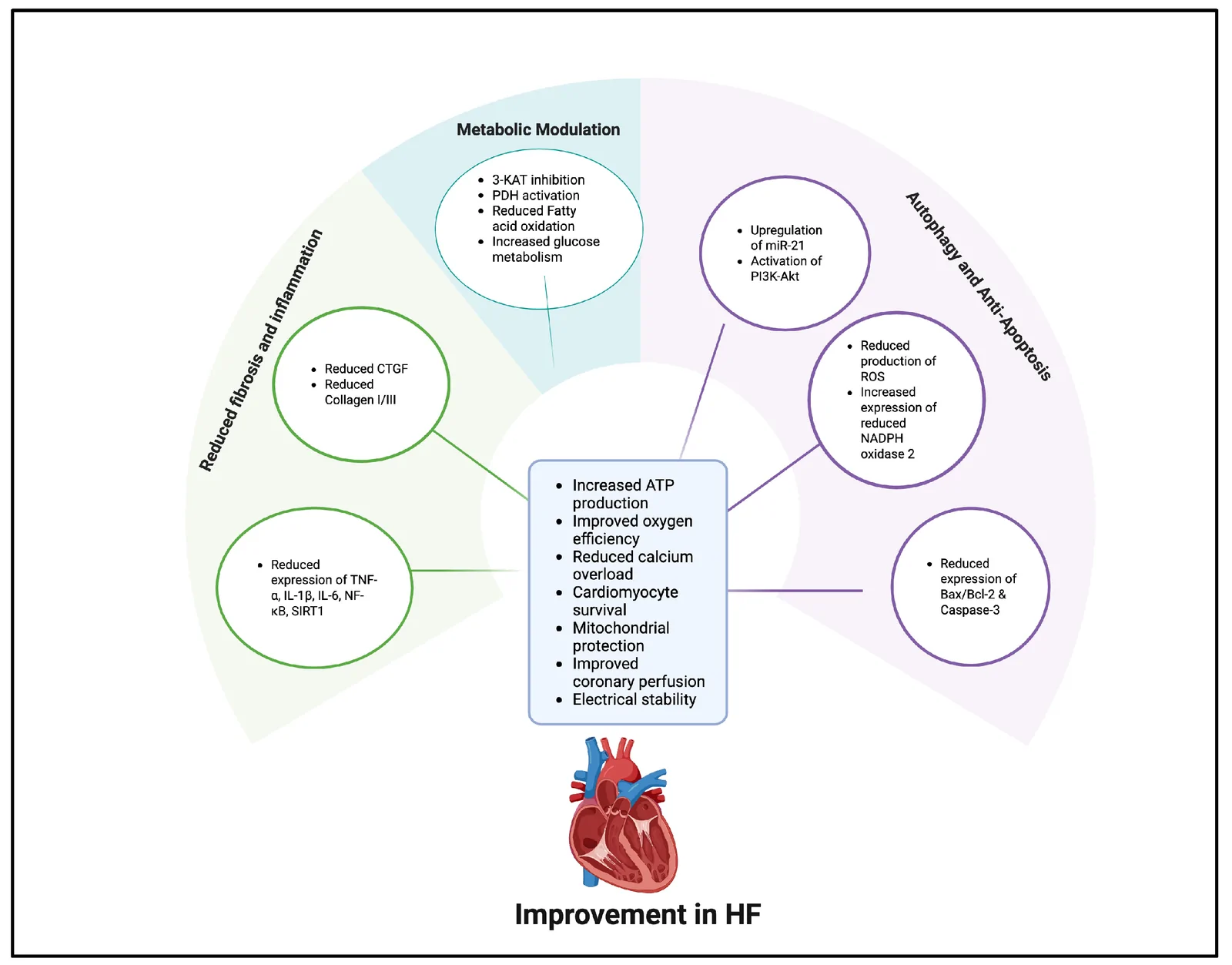
Multidimensional mechanisms through which trimetazidine helps improve cardiac function in heart failure. Abbreviations: 3-KAT: Long-chain 3-ketoacyl-CoA thiolase; Akt: Protein kinase B; ATP: Adenosine triphosphate; CTGF: Connective tissue growth factor; HF: Heart failure; IL: Interleukin; miR-21: MicroRNA-21; NF-κB: Nuclear factor-kappa B; NADPH: Nicotinamide adenine dinucleotide phosphate; PDH: Pyruvate dehydrogenase; PI3K: Phosphoinositide 3-kinase; ROS: Reactive oxygen species; SIRT1: Sirtuin-1; TNF-α: Tumor necrosis factor-alpha.

Trimetazidine may also attenuate adverse ventricular remodeling by reducing inflammation, oxidative injury, and myocardial fibrosis. Experimental evidence suggests that preservation of mitochondrial function and improved energy production help maintain calcium homeostasis, thereby reducing calcium overload, improving excitation–contraction coupling, and limiting progressive myocardial dysfunction. Collectively, these metabolic, antioxidant, anti-apoptotic, and anti-remodeling effects provide the mechanistic basis for the improvements in ventricular function, exercise capacity, and quality of life observed in patients with heart failure (Figure 1).

## 5. Clinical Evidence: Efficacy of Trimetazidine in Heart Failure

### 5.1. Randomized Controlled Trials

Several randomized trials have investigated the safety and efficacy of Trimetazidine (40/60–70 mg/day) in patients (N = 1287) with confirmed diagnosis of heart failure of ischemic (N = 1104) and non-ischemic (N = 183) etiology (Table 1). The patients included in these trials were spread across NYHA classes I to IV based on the functional capacity or development of symptoms on physical activity and objective evidence of cardiovascular disease. The patients in all but one trial (LVEF < 55%) were diagnosed as heart failure with reduced ejection fraction (HFrEF- LVEF ≤ 40%). The duration of trials ranged from 1 to 24/48 months. The patients were managed with conventional therapy prior to trial enrolment and were randomized to either trimetazidine or placebo.

The studies evaluated and estimated the effect of trimetazidine on various efficacy parameters using objective and subjective tests. Patients underwent echocardiography to record various 2D Echocardiographic and Doppler indices, including left ventricular end-diastolic dimensions and volumes (LVEDD, LVEDV), left ventricular end-systolic dimensions and volumes (LVESD, LVESV), LV ejection fraction (LVEF), and stroke volume (SV). The LV wall stress (LVESS) and left ventricular (LV) mass (LVM) was calculated. The ratio of transmitral flow velocities (peak early: E wave and late: A wave) and their ratio (E/A) were also recorded. Functional exercise capacity was estimated using the 6 min walk distance (6MWD). The left ventricular dysfunction 36 questionnaire (LVD-36) was used to measure the impact of LV dysfunction on daily life and well-being. Some of the studies also determined the effect of trimetazidine on systemic oxidative stress, which are markers of pathological abnormalities to lipids, cardiac, and vascular tissues.

#### 5.1.1. Echocardiographic Parameters

##### Effect on LVEF

**Figure 2:**
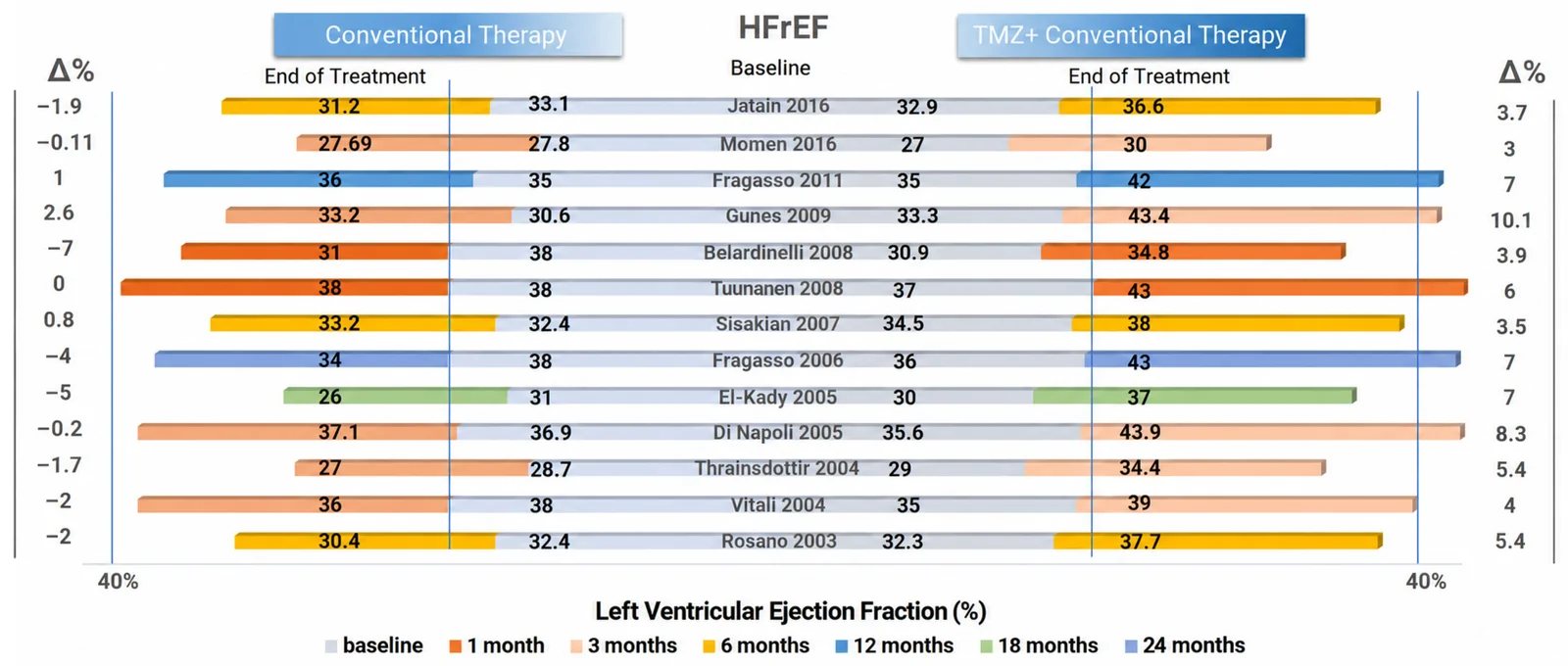
Effect on trimetazidine as an add-on to conventional therapy on LVEF versus conventional therapy alone across RCTs of 1-, 3-, 6-, 12-,18-, and 24-month duration [12,14,16,17,18,19,20,21,22,23,24,25,26]. TMZ: Trimetazidine; HFrEF: Heart Failure with reduced Ejection Fraction.

Patients on trimetazidine in addition to conventional therapy had a consistent and sustained improvement in LVEF. Across studies, the increase in LVEF was significantly greater in the trimetazidine-treated patients than patients treated with conventional therapy alone, especially in long-term studies. The improvement in LVEF observed in studies of short duration (1–6 months: 3.9 to 5.4–10.1%) was also confirmed in longer duration studies (6–24 months: 5.4% to 7%). The increment [median (min-max)] in LVEF was ∆5.4% (3% to 10.1%) with trimetazidine, while there was a progressive deterioration in the conventional therapy group ∆−1.7% (−7% to 2.6%) with duration (Figure 2). Among the 13 studies that reported baseline and post-treatment LVEF values in patients with HFrEF, 5 studies demonstrated improvement in the severity of left ventricular dysfunction class. As per the American College of Cardiology (ACC) classification, the LVEF improved from moderate to mild LV dysfunction (increased from ≤40% to >40%).

Subgroup analysis revealed that regardless of etiology, there was no significant difference in the LVEF increment in patients treated with trimetazidine between ischemic and non-ischemic patients (8.6% ± 3.6% vs. 10.2% ± 5.1%, *p* = 0.203). However, in a subgroup analysis of patients with HF, an increase in LVEF with trimetazidine was found to significantly correlate with the presence of diabetes (*r* = 0.524, *p* < 0.001). In the trimetazidine-treated group, the LVEF increased significantly more in those with than without diabetes (12.8% ± 4.5% vs. 7.7% ± 3.1, *p* < 0.001). Thus, it can be argued that trimetazidine as an add-on therapy to existing conventional therapy in patients with heart failure, especially for those who are diabetic, may improve LV and RV functions [19].

##### Ventricular Volume

**Figure 3:**
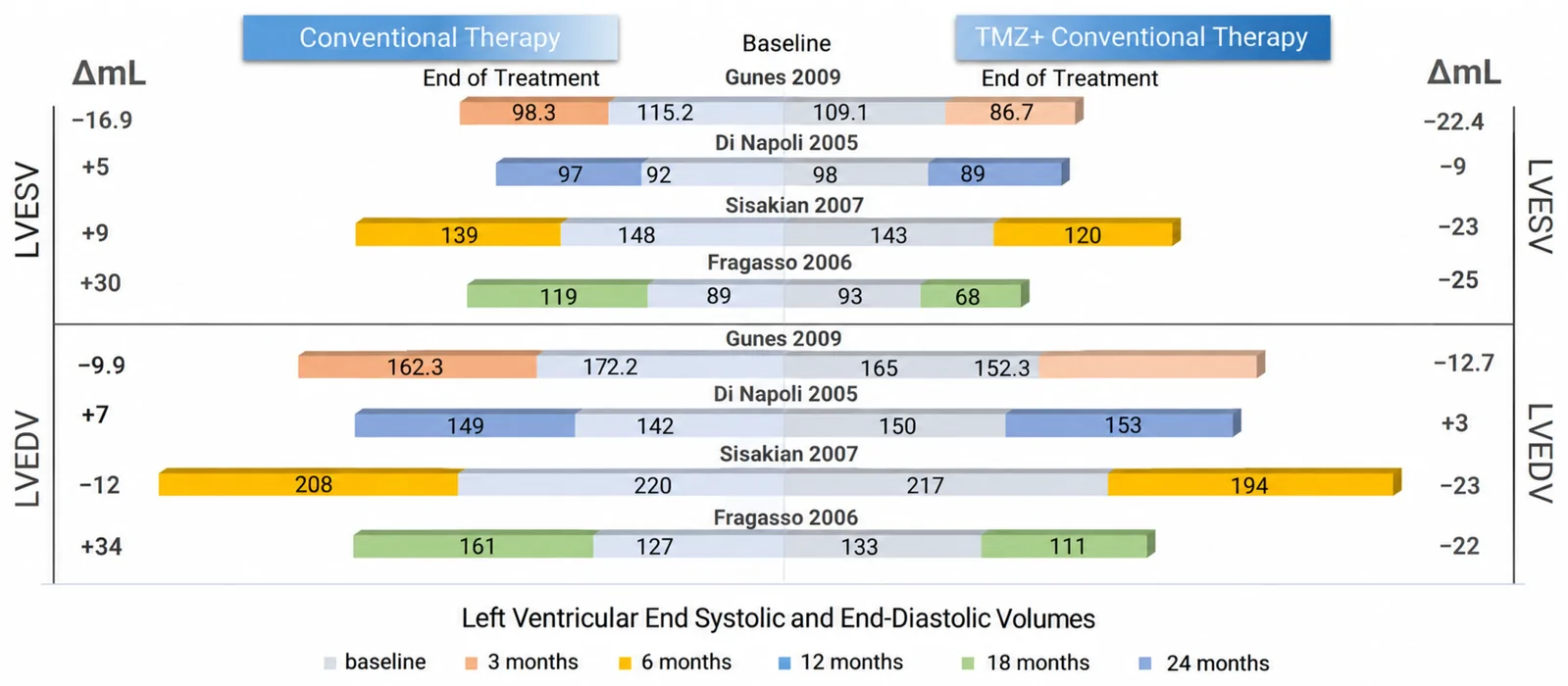
Effect of trimetazidine as an add-on to conventional therapy on LV Volumes (surrogate for LVEF) versus conventional therapy alone across RCTs of 6-, 12-, 18-, and 24-month duration [14,17,19,23]. TMZ: Trimetazidine; LVEDV: Left Ventricular End-Diastolic Volume; LVESV: Left Ventricular End-Systolic Volume.

All of the studies that reported a change in LV volumes reported a significant decrease over baseline in the trimetazidine-treated group versus the conventional therapy group alone (Figure 3). The median decrease in LVESV was 17.7 mL (−25 mL to −9 mL), and LVEDV was 17.65 mL (−23 mL to +3 mL) in the trimetazidine-treated group compared to an increase of median 9 mL (+5 mL to +30 mL) and 7 mL (−12 mL to +34 mL) in the conventional treatment group. Thus, an increase in LVEF in the patients treated with trimetazidine resulted in a significant decrease in LV volumes. In contrast, the LV volumes progressively increased in the conventional therapy group from baseline up to 24 months [14,23]. The differences in LVESV and LVDEV were significant across the various study periods in the trimetazidine group compared to baseline and conventional treatment group [14,23].

The other left ventricular performance indicators determined were left ventricular end-diastolic volume index (LVEDVI) and end-systolic volume index (LVESVI) in a 6-month study. A significant decrease in LVEDVI (−3.7 ± 1.9 mL/m^2^, *p* < 0.04 compared to baseline and placebo) and LVESVI (–2.2 ± 1.1 mL/m^2^, *p* < 0.04 compared to placebo and baseline) was noted in patients treated with trimetazidine, while an increase in LVEDVI (1.9 ± 1.1 mL/m^2^) and LVESVI (0.48 ± 0.4 mL/m^2^) was found in the placebo group [22].

##### Effect on LV Contractility (SWTI)

Contractile dysfunction in moderate heart failure may be attributed to fatty acid oxidation [12]. Trimetazidine, which acts on the fatty acid oxidation, improves contractility of dysfunctional myocardium in chronic ischemic heart disease. A 3-month study evaluating the effect on systolic wall thickening index score (SWTI score) found a significant reduction in left ventricular wall thickness in trimetazidine-treated patients (from 2.2 ± 0.8 to 1.7 ± 0.9, *p* < 0.05) [12].

The left ventricular global longitudinal strain (LV GLS) parameter also represents LV contractile function. In patients with HFrEF, there was a significant improvement in GLS in the trimetazidine group (−6.9% ± 2.4% to −8.4% ± 2.6%, *p* = 0.000), whereas no significant change occurred in the control group (1.5% + 0.9% vs. −0.7% + 1.7%, *p* = 0.001) [29].

##### Effect on Wall Motion Score Index

**Figure 4:**
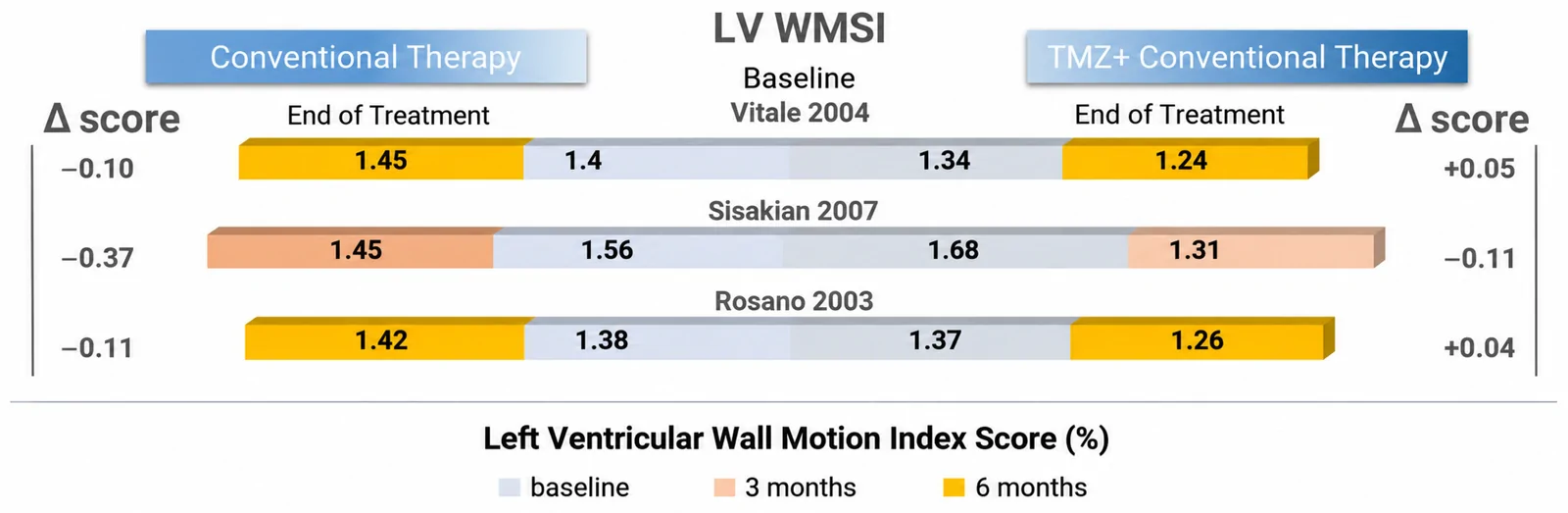
Effect on trimetazidine as an add-on to conventional therapy on left ventricular wall motion score index (WMSI) versus conventional therapy alone across RCTs of 3- and 6-month duration. The change in the score from baseline (Δ score) was the primary end point and denoted at both ends of each study [22,23,26].

The wall motion score index (WMSI) significantly decreased in patients treated with trimetazidine from a median 1.37 (1.34−1.68) to 1.26 (1.24–1.31), while it slightly increased from 1.4 (1.38–1.56) to 1.45 (1.42–1.45) in the conventional therapy group (Figure 4) [22,23,26].

##### Qualitative Assessment of Both Systolic and Diastolic Regional Ventricular Function

**Figure 5:**
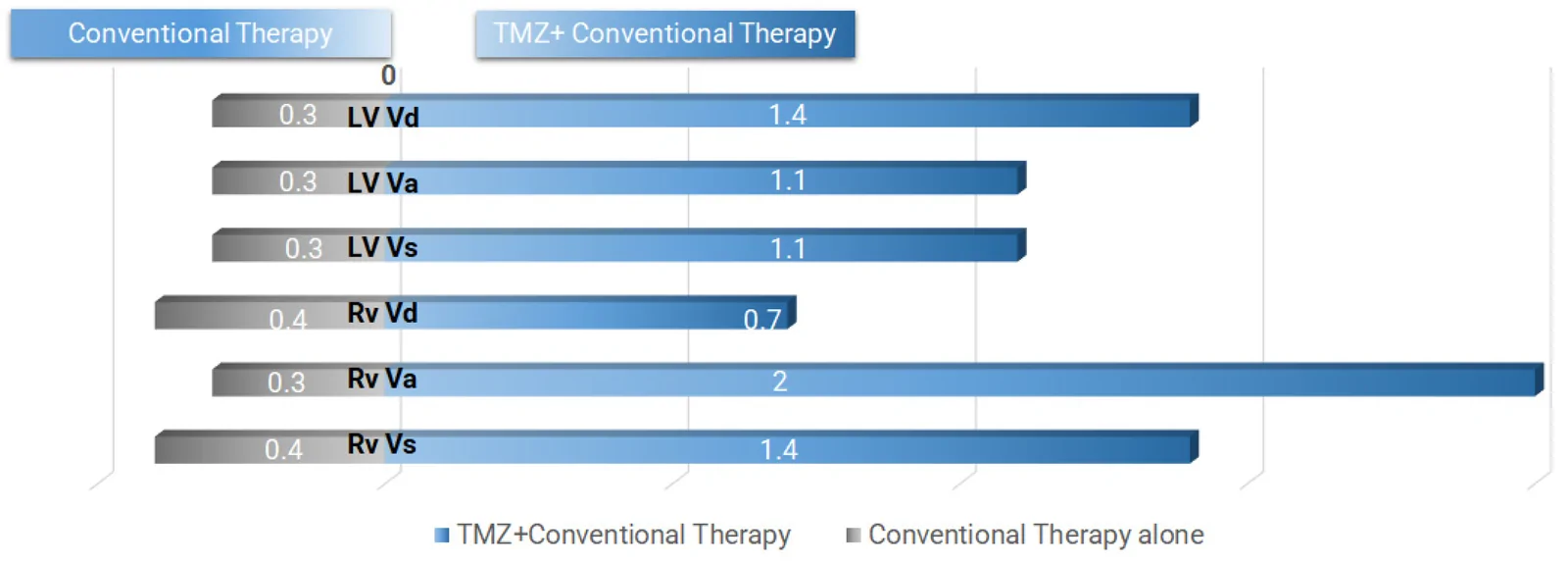
Comparison of changes in mean myocardial velocities between patients treated with trimetazidine and placebo [19]. LV: left ventricle; RV: right ventricle; TMZ: Trimetazidine; Vd, Va, and Vs: early and late diastolic, and systolic myocardial velocities.

Tissue Doppler imaging, which qualitatively assesses both systolic and diastolic regional ventricular function, found significant improvements in left and right ventricular myocardial velocities with trimetazidine in both early and late diastolic and systolic phases compared to conventional therapy alone in a 3-month study (Figure 5) [19]. The improved myocardial velocities may mirror early positive remodeling, and perhaps the chronic benefits to clinical status, such as improved exercise capacity, may be attributed to these enhancements. Interestingly, the positive effects of trimetazidine on right ventricular imaging parameters may be associated with the functional improvement of heart failure patients [19].

Left ventricular diastolic function, evaluated through the E/A ratio on mitral flow, significantly improved in patients treated with trimetazidine in two studies (from 0.68 ± 0.1 to 0.89 ± 0.3 and 0.58 ± 0.11 to 0.87 ± 0.10), whereas it remained unchanged in patients treated with the conventional therapy group [22,26]. Additionally, trimetazidine was associated with a significant decrease in the E/e’ ratio (from 10.7 ± 4.5 to 8.7 ± 2.2; *p* < 0.05), whereas in the conventional therapy group, it remained unchanged (from 13.6 ± 5.3 to 12.9 ± 5.8, *p* = >0.05). An increased ratio of early transmitral flow velocity to early diastolic velocity of the mitral annulus (E/e’) obtained by tissue doppler imaging has been linked with LV filling pressure and is a powerful predictor of negative clinical outcome [19]. Thus, the reduced E/e’ ratio may lead to improved clinical outcomes with trimetazidine, noted in long-term studies.

#### 5.1.2. Change in NYHA Functional Class

**Figure 6:**
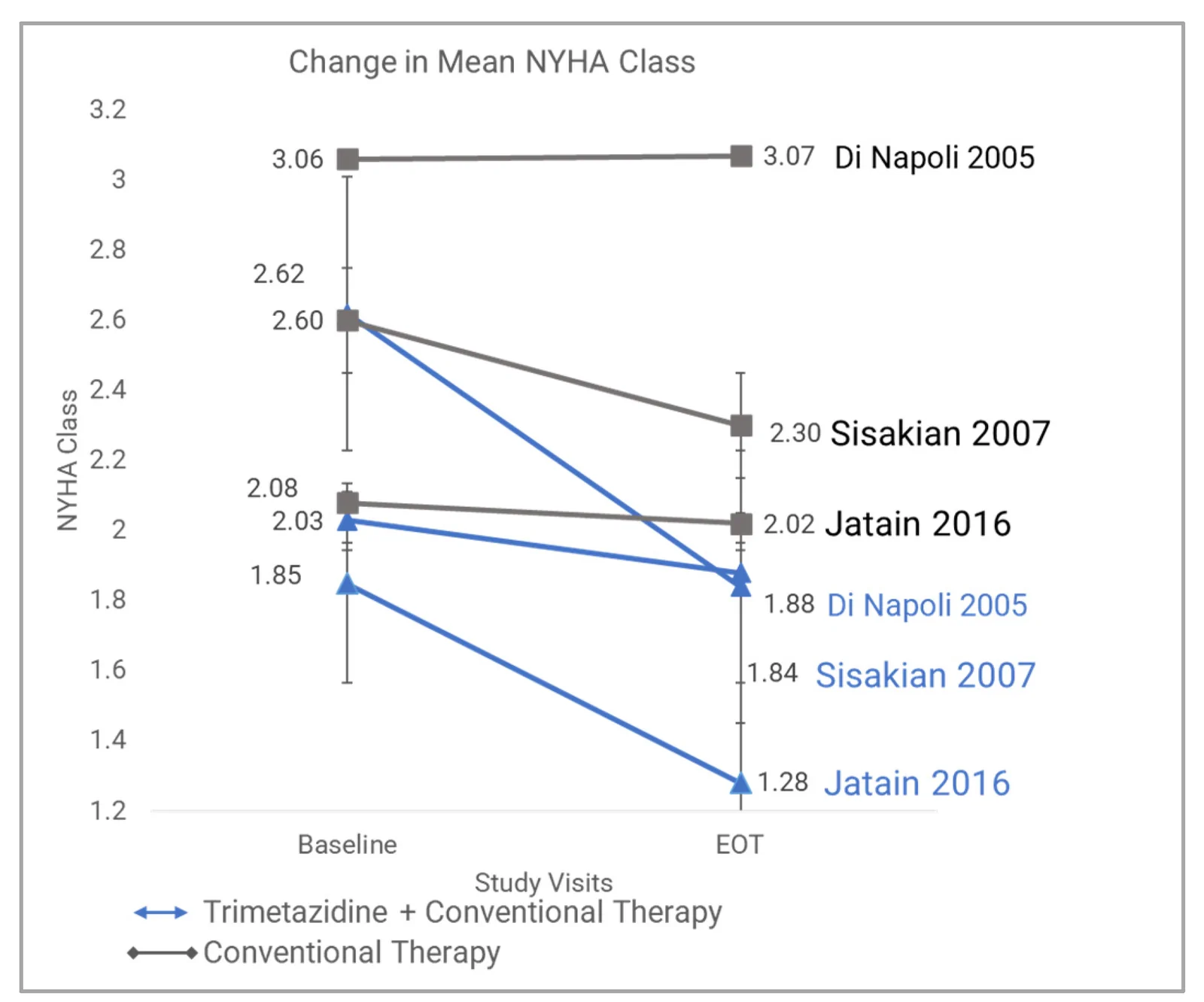
Change in mean NYHA Class: Trimetazidine + Conventional therapy vs. Conventional therapy alone [14,20,23]. NYHA: New York Heart Association. EOT–End of study.

Seven studies reported changes in the patients’ functional status (NYHA class) during treatment with trimetazidine. At the end of 3, 12, and 18 months, a significant functional improvement was noted in most patients receiving trimetazidine as an add-on to existing conventional therapy (Figure 6) [14,20,23].

In the trimetazidine group, patients in NYHA functional class III decreased, while patients in NYHA classes II and I increased compared to baseline, indicating an improvement of 1 to 2 grades. In contrast, the conventional therapy group showed a significant increase in patients in NYHA classes IV and III compared to the trimetazidine group (Table 2) [17,20,26].

#### 5.1.3. Effect on Exercise Capacity

**Figure 7:**
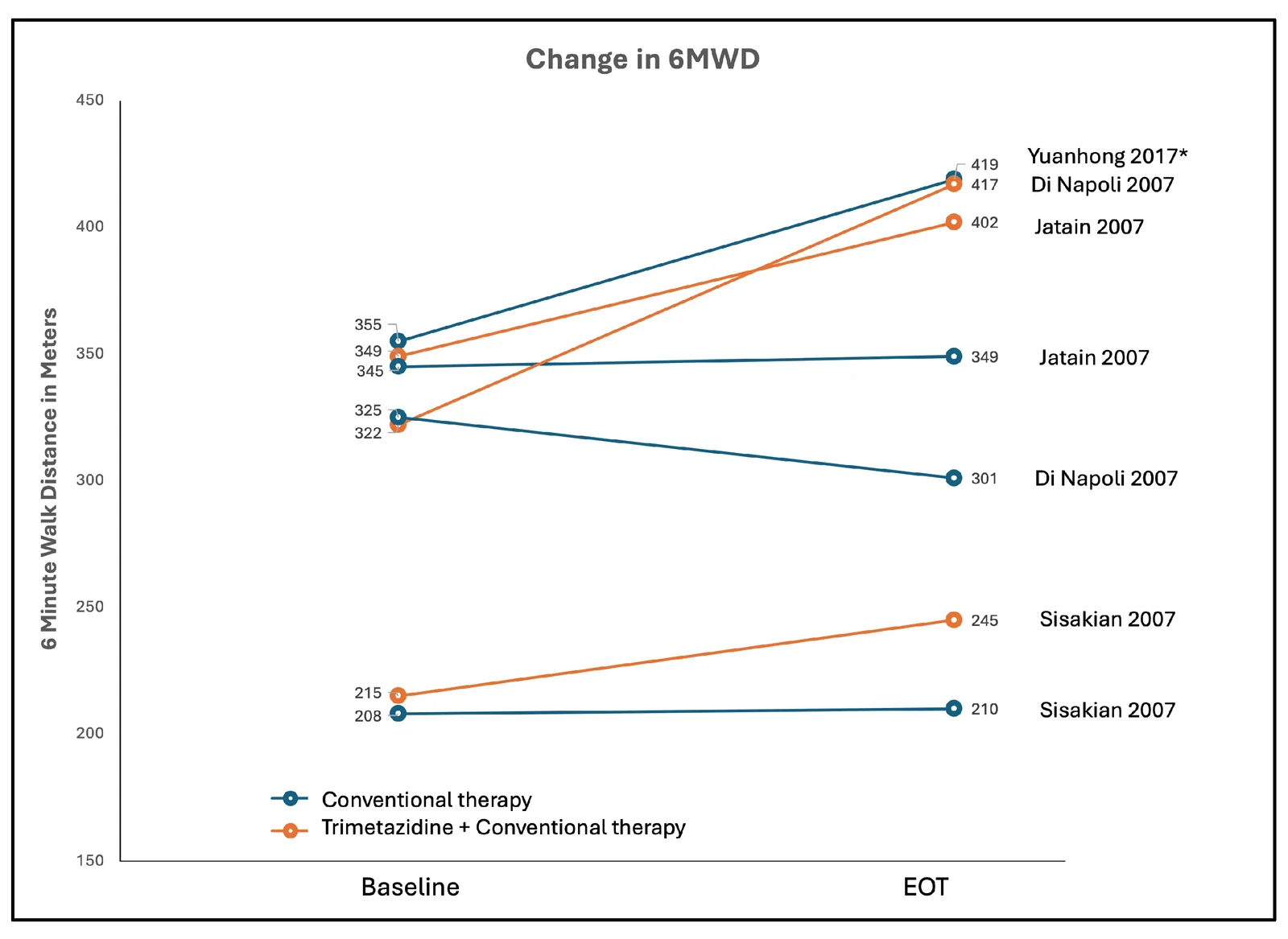
Change in mean 6-MWD: trimetazidine/Conventional therapy vs. Conventional therapy alone [15,20,23,27]. * The Yuanhong study (2017) had no control arm. 6-MWD: 6-Minute Walk Distance, EOT–End of study.

The 6 min walk distance (6MWD) showed significant improvement in the trimetazidine group from baseline to the end of follow-up [15]. The distance walked decreased in the placebo group at various study visits across studies. The difference in mean distance walked at the end of follow-up was statistically significant in favour of trimetazidine (Figure 7) [15,20,23,27].

Apart from the 6MWT, the duration of peak exercise also improved with trimetazidine in both short- and long-duration studies. The duration of peak exercise was significantly greater with trimetazidine than with placebo (75 ± 25 s vs. 25 ± 5 s, *p* < 0.01) in the 24-month study. Accordingly, the mean maximum work at peak exercise improved by 1.2 metabolic equivalents with trimetazidine. Consistently, the improvement in mean maximum work at peak exercise in the trimetazidine group was significantly greater than that in the conventional therapy group (2 vs. 0.1 metabolic equivalents, *p* < 0.001) [16]. The total exercise time also improved in trimetazidine (440 ± 140 s to 530 ± 145 s), while in control it decreased (465 ± 130 to 458 ± 132 s) in the 3-month study [12]. Belardinelli et al., 2007 reported significant improvement in cardiopulmonary exercise capacity, peak VO_2_, anaerobic threshold, ventilation, and O_2_ pulse versus placebo without change in heart rate at the end of 1 month [11]. Thus, it is rational to accept that trimetazidine has a protective action on cardiac function, which corresponds to improvement in exercise endurance.

#### 5.1.4. Electrophysiological Effects

In a single 6-month study, the electrophysiological effects of trimetazidine were assessed in patients with chronic heart failure (CHF). Trimetazidine effectively reduced Tpeak–Tend and Tpeak, which is a non-invasive marker of dispersion of ventricular repolarization [13]. In another 1-month study, trimetazidine significantly decreased the QTc interval by >20 milliseconds in 64% of patients, while in the conventional therapy group, only 15% of patients demonstrated a decrease (*p* = 0.002). The effect of trimetazidine on QTC may be indicative of its modulatory action on sympatho-vagal balance, anti-inflammatory properties, and reduction of endothelin-1, as demonstrated in experimental studies [28]. This beneficial effect, though evident, needs confirmation in a larger patient population to suggest the use of trimetazidine for life-threatening arrhythmias resulting in HF.

#### 5.1.5. Effect on Contractile Performance of Skeletal and Cardiac Muscle

Trimetazidine offers a unique therapeutic approach in heart failure by targeting metabolic abnormalities in skeletal and respiratory muscles, which contribute significantly to symptoms like dyspnea and fatigue [5,17]. By shifting energy production from fatty acid oxidation to glucose oxidation, trimetazidine enhances the efficiency of muscle function, thereby improving exercise capacity and reducing HF symptoms. Its ability to boost contractile performance in these muscles not only alleviates the classic symptoms of HF, but also highlights its relevance in improving patients’ quality of life [5,18]. However, trimetazidine’s muscle-enhancing effects have led to its inclusion on the International Olympic Committee’s list of prohibited substances (S4 list) due to its performance-enhancing potential since 2014.

#### 5.1.6. Clinical Benefits of Pleiotropic Actions

##### Systemic Oxidative Markers

There is ample clinical evidence to show that free radicals are increased in CHF, with some studies reporting almost a four-fold increase compared to healthy controls [30]. It is well-established that free radicals generated under conditions of oxidant stress can result in pathological modification of lipids, proteins, and tissues in the blood vessels. An antioxidant effect of trimetazidine was indicated by a reduction in systemic oxidant stress markers, such as MDA and LOOHs. Belardinelli et al. (2007) demonstrated a significant decrease in both plasma MDA levels (from 3.98 ± 0.69 to 2.15 ± 0.59 mmol/L) and LOOH levels (from 3.72 ± 0.9 to 2.06 ± 0.6 mmol/L) compared with the placebo (*p* < 0.001 for both vs. placebo) [11]. Thus, this study confirmed that trimetazidine improved the endothelial vasomotor function in CHF, which may be attributed to its antioxidant potential [11]. Additionally, the increase in radial artery diameter in response to acetylcholine was more evident in patients with two or more coronary risk factors with higher plasma levels of free radicals than in patients with one or no risk factors [11]. Thus, the antioxidant action of trimetazidine may improve the endothelial vasomotor function [11]. The antioxidant and subsequent vasodilatory action results in elevated stroke volume during daily submaximal physical activities that can aid in improving functional capacity and thereby quality of life by enhancing an active lifestyle [11].

##### Prognostic Markers BNP, cTnT

**Figure 8:**
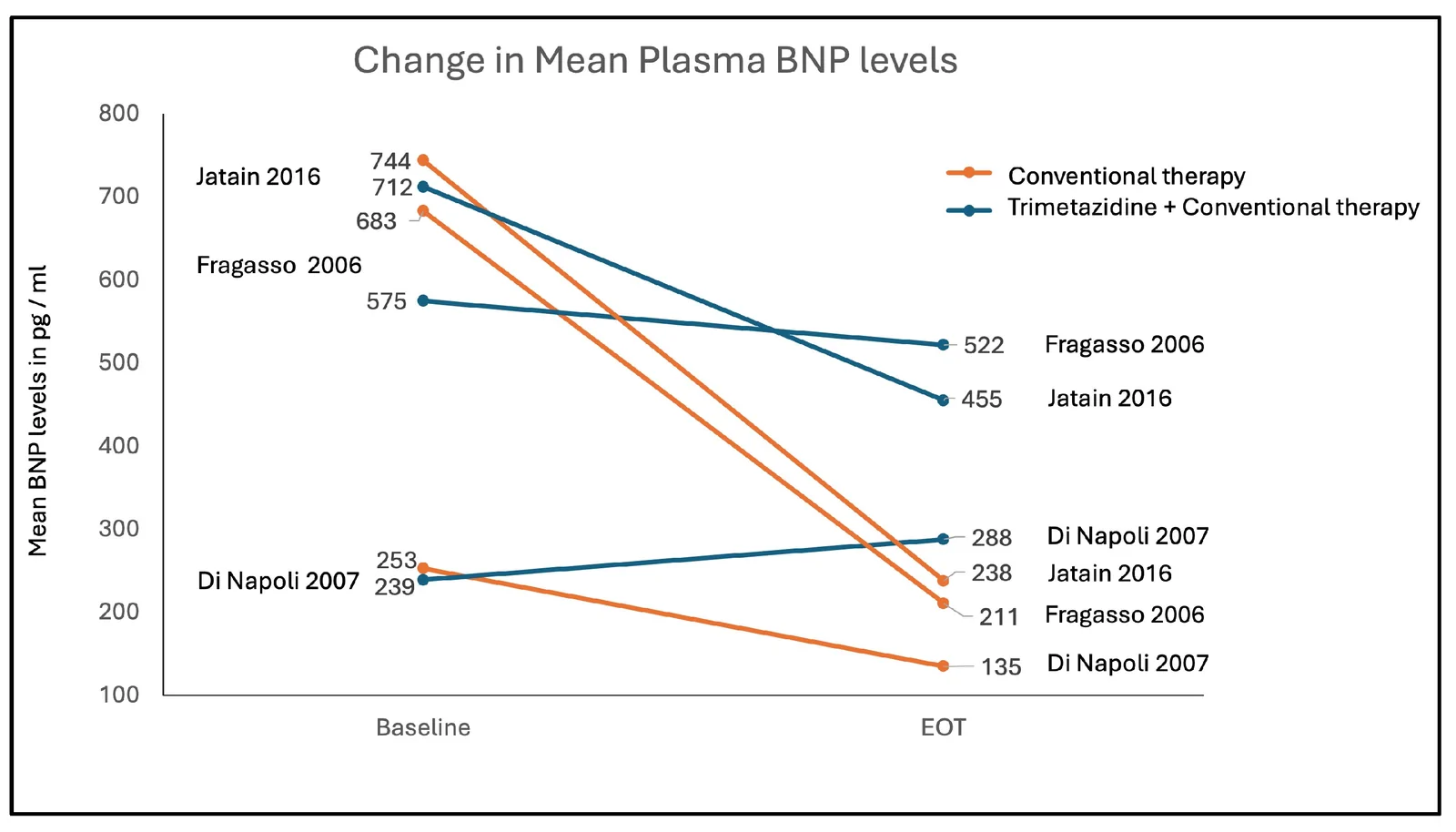
Change in plasma BNP levels: Trimetazidine vs. Placebo [15,17,20]. BNP: Brain Natriuretic Peptide; EOT–End of study.

BNP, NT-pro-BNP, and cTnT are markers of myocardial load and myocyte injury. Studies assessing the effect of trimetazidine in patients with heart failure treatment found significant reductions in these markers, paralleled by clinical and echocardiographic improvements (Figure 8 and Table 3) [15,20]. These data suggest that trimetazidine may modulate neurohormonal pathways in patients with ischemic cardiomyopathy and thus reduce the cellular damage that typifies the chronic progression of heart failure and left ventricular remodeling [15].

##### Other Biomarkers: CRP

CRP is a marker of systemic inflammation and found to be elevated in patients with heart failure [31,32]. Higher levels of CRP correspond to characteristics of more severe heart failure and are linked to mortality and morbidity [33]. Therefore, reduction of CRP may be of therapeutic and prognostic importance [31]. Clinical studies that evaluated the effect of trimetazidine found that CRP either reduced (by 38% from 2.6 ± 0.9 to 1.6 ± 1.0) in short-term studies or remained stable in long-term studies (2.5 ± 1.0 to 3.0 ± 2.0 mg/L), while the control group increased (2.4 ± 1 to 7.0 ± 5.0 mg/L) [12,15]. These effects suggest improvement in endothelium-dependent vasodilatation, which is of mechanistic importance, especially in heart failure with ischemic etiology.

#### 5.1.7. Effect on Cardiac Hospitalization and Mortality

Although the primary evidence supporting trimetazidine in heart failure comes from improvements in surrogate markers such as left ventricular function, exercise capacity, and biomarkers, several studies have also evaluated its impact on major clinical outcomes, including hospitalization and mortality. However, these findings should be interpreted with caution, as the available evidence is derived predominantly from relatively small, single-center randomized trials with limited statistical power, and only a few studies were specifically designed to evaluate hard clinical endpoints. Consequently, while the available data are encouraging, larger multicenter randomized controlled trials are required to establish the effect of trimetazidine on long-term clinical outcomes.

**Figure 9:**
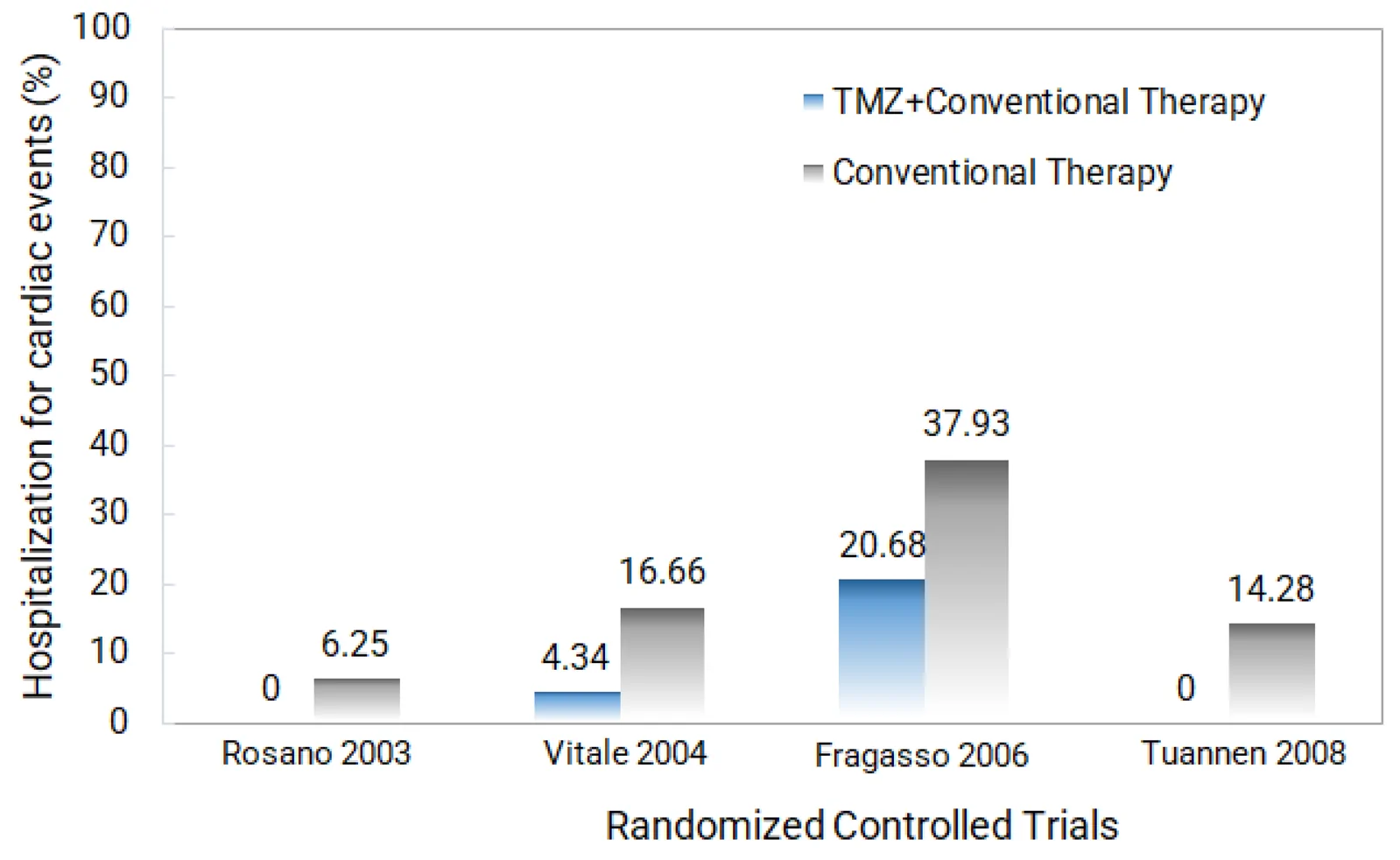
Effect of trimetazidine on hospitalization for cardiac events [17,22,25,26]. TMZ: Trimetazidine.

Four studies reported the effect of trimetazidine on the hospitalization events in patients with heart failure. Among these studies, significantly greater hospitalization events were reported with the conventional therapy group than the group treated with add-on trimetazidine. Overall, ~9% needed hospitalization in the trimetazidine therapy group compared to ~22% in the conventional therapy group for cardiac causes (Figure 9) [34].

**Figure 10:**
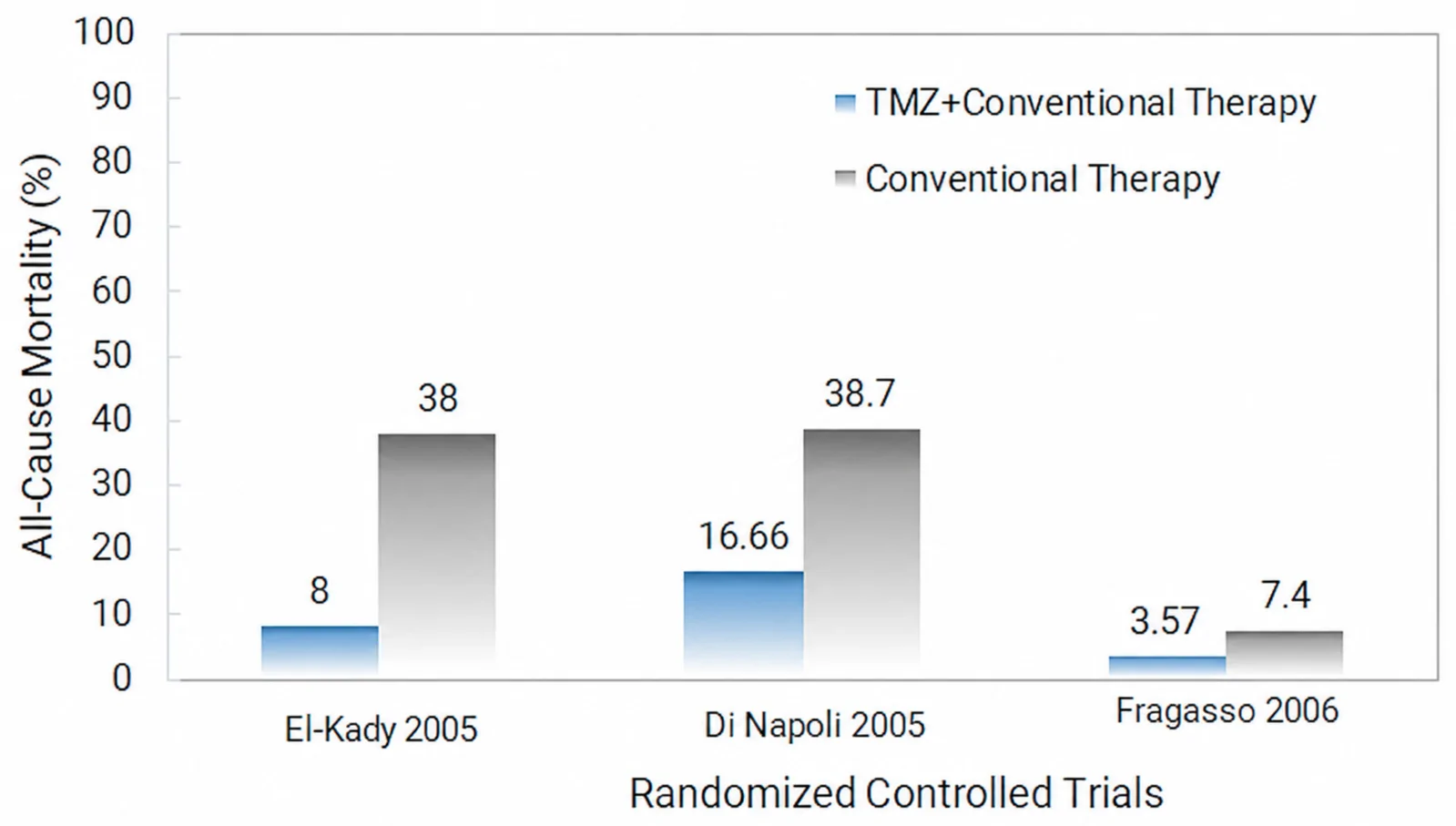
Effect of trimetazidine on all-cause mortality [14,16,17]. TMZ: Trimetazidine.

The effect on mortality was also reported by these studies, along with a 4-year follow-up extension of the Di Napoli study in 2005 (Figure 10). Trimetazidine, when added to the conventional therapy, significantly reduced all-cause mortality in one study, while the pooled analysis of all three studies also found a significant reduction [35].

In a retrospective study by Fragasso et al., 2013, trimetazidine add-on improved the 5-year survival by 11.3%, reduced cardiovascular mortality by 8.7%, and hospitalization for any cardiovascular event by 11.4% [36].

Overall, the available evidence suggests that trimetazidine, when used as an adjunct to guideline-directed medical therapy, may reduce cardiovascular hospitalization and potentially improve survival in selected patients with HFrEF. Nevertheless, these findings are based largely on small clinical studies and pooled analyses rather than adequately powered outcome trials. Therefore, the observed reductions in hospitalization and mortality should be considered hypothesis-generating and require confirmation in contemporary multicenter randomized controlled trials before definitive conclusions can be drawn.

#### 5.1.8. Quality of Life

The Left Ventricular Dysfunction Questionnaire (LVD-36), which measures the impact of left ventricular dysfunction on daily life and well-being, found that the LVD-36 score improved with trimetazidine. LVD-36 score consistently decreased with trimetazidine (at 3 months from 25.5 ± 4.99 to 21.03 ± 4.6, *p* = 0.001: at 6 months 21.03 ± 4.6 to 16.14 ± 5.15, *p* = 0.001; at 13 months from 18.8 ± 7.0 to 15.1 ± 6.3; *p* = 0.038) and quality of life score increased (from 63 ± 19% to 71 ± 15%; *p* = 0.07), whereas in the conventional therapy group, LVD-36 score increased or remained unchanged (at 3 months 24.4 ± 4.1 to 24.14 ± 3.14, *p* = 0.225, at 6 months no change, at 13 months from 18.4 ± 8.8 to 19.2 ± 9.5; *p* = 0.07) and QoL score decreased (from 66 ± 12% to 61 ± 20%; *p* > 0.05; *p* = 0.04 vs. trimetazidine) [17,19].

Another 6-month study evaluating QoL using the visual analogue scale found significant improvement in the trimetazidine-treated patients (increased from 4.1 to 6.3; *p* < 0.01), while the conventional groups showed no change [26].

### 5.2. Meta-Analyses

Hitherto, six meta-analyses have been performed, with three of these comprising RCTs in patients with heart failure due to both ischemic and non-ischemic etiology. Among the other three, two comprised patients with ischemic etiology, and a single one of patients with non-ischemic etiology (Table 4).

The studies that comprised both ischemic and non-ischemic HF patients aimed to explore the benefits of trimetazidine as an add-on to conventional therapy in terms of improvement in symptoms, cardiac function, and overall impact on clinical outcomes, along with effects on LV remodeling.

In agreement with the findings of the individual RCTs described previously, patients treated with trimetazidine demonstrated significant improvements in LVEF, TED time, a decrease in NYHA class, and a decrease in left ventricular dimensions, accompanied by underlying improvements in BNP levels. These benefits reflected as a reduction in hospitalization for cardiac causes (RR: 0.43, *p* = 0.03) and all-cause mortality [34,38,39].

While major studies included in these meta-analyses remained similar until the year of their publication, a few endpoints analyzed differed among them. Therefore, the effects of trimetazidine on the left ventricular structure and functions and, consequently, improvement in the NYHA class were found to be significant in all three meta-analyses. In addition to the effect of trimetazidine on reducing hospitalization, it was also significant in both meta-analyses by Gao et al. and Zhou (2014) [34,40]. While Gao et al. did find a significant reduction in ACM, Zhou et al. (2014) did not (Table 5) [34,40].

Meta-analyses that included studies with only patients with Ischemic HF found an improvement in LVEF, a significant improvement in 6-MWD and NYHA class. These meta-analyses also found a significant reduction in All-Cause Mortality (ACM). A meta-analysis of studies involving non-ischemic heart failure found significant improvements in LVEF and 6-MWD. Both of these improvements were greater than those found in the meta-analysis of studies in patients with ischemic heart failure. Significant improvement was also noted in the non-ischemic HF meta-analysis in the left ventricular systolic volume as well. Since Garjek et al., 2015 only aimed to assess the effect of trimetazidine on ACM, it included only three RCTs that reported it (Table 6) [35].

Thus, consistent with the several RCT findings, the meta-analyses confirmed the benefits of trimetazidine as an add-on to the conventional therapy in patients with both ischemic and non-ischemic heart failure.

## 6. Personalised Approach in HF Management

Based on extensive clinical evidence, the ESC 2021 recommends trimetazidine for Heart Failure with Reduced Ejection Fraction (HFrEF) [42]. According to the 2021 ESC Guidelines for the diagnosis and treatment of acute and chronic heart failure, trimetazidine is recommended as a second-line add-on in patients with HFrEF despite the use of a beta-blocker in patients with a heart rate of <70 bpm (Class II).

Among the various factors that guide the pharmacotherapy of heart failure, the primary drivers are LVEF, etiological factors, and comorbid conditions. The clinical benefits of pharmacotherapy have been typically restricted to patients with HFrEF (EF ≤ 40%), while effects in Heart Failure with Preserved Ejection fraction (HFpEF) are so far inadequate or limited. This indicates the necessity to personalize pharmacotherapy based on causal primary factors [43]. The leading etiological factors for heart failure include ischemic heart disease and hypertensive heart disease in one-fourth of the patients, followed by cardiomyopathy in ~6.5%, mitral valve disease, alcoholic cardiomyopathy, aortic valve disease, and myocarditis in <3% patients [44]. Based on these parameters, the treatment of heart failure varies, though some pharmacological agents will be common to most cases such as diuretics, vasodilators, RAAS blockers, and metabolic modulators. The manner in which these agents are used and the exactitudes of individual etiology, type every patient individually, thus necessitating personalized therapy [45]. Metabolic modulators are an important add-on option for the treatment of heart failure with reduced ejection fraction in ischemic heart failure and ischemic dilated cardiomyopathy. The majority of studies have confirmed the clinical benefits of trimetazidine in HFrEF, regardless of ischemic or non-ischemic etiology. Specifically, various studies have confirmed the benefits of trimetazidine in patients with non-ischemic heart failure, such as diabetic cardiomyopathy and ischemic dilated cardiomyopathy. Subgroup analyses have demonstrated that trimetazidine has better efficacy in patients with both ischemia and diabetes than in those without diabetes.

## 7. Recent Advances and Future Directions

In recent years, the treatment of heart failure has made significant advances, with the advent of evidence-based therapies such as angiotensin receptor-neprilysin inhibitors (ARNIs), sodium-glucose cotransporter-2 (SGLT2) inhibitors, mineralocorticoid receptor antagonists, and cardiac myosin activators, which have led to significant improvements in clinical outcomes [46]. These therapies are primarily directed at neurohormonal activation, ventricular remodeling, renal protection, and myocardial contractility. However, a significant proportion of patients have ongoing symptoms, reduced exercise capacity, frequent rehospitalizations, and progressive ventricular dysfunction, indicating that important pathophysiologic mechanisms, including metabolic abnormalities, are not adequately addressed.

Istaroxime is a cardiotonic steroid that acts by stimulation of SERCA2a and suppression of NKA activity. It not only has inotropic actions, but also demonstrates lusitropic positive effect and is better tolerated than existing inotropic drugs in various HF trials- HORIZON-HF (Hemodynamic, Echocardiographic, and Neurohormonal Effects of Istaroxime, a Novel Intravenous Inotropic and Lusitropic Agent), The Clinical Study of the Safety and Efficacy of Istaroxime in Treatment of ADHF [47]. However, due to its short half-life, research is focused on developing newer derivatives with longer half-lives. Additionally, long-term safety and efficacy data are also being evaluated [48]. Another drug, Omecamtiv Mecarbil, a cardiac myosin activator, has completed the phase III trial GALACTIC-HF. However, it demonstrated limited clinical benefit in terms of reduction in the composite end point of time to first HF event or CV death among patients with severe HF [48,49]. It also offered no evidence of improved survival or quality of life. This drug therefore did not receive regulatory approval. Elamipretide or Bendavia is a small mitochondrially-targeted tetrapeptide that apparently attenuates the formation of toxic reactive oxygen species and stabilizes cardiolipin [50]. In the Phase II study, this drug showed a reduction in left ventricular diastolic and systolic volume; however, long-term safety and efficacy need to be evaluated in larger studies [51].

The advent of SGLT2 inhibitors has re-invigorated interest in myocardial metabolism as a therapeutic target. SGLT2 inhibitors indirectly modulate systemic energy metabolism through increased ketone body utilization and an improvement in myocardial energetics. Trimetazidine has a direct effect at the cellular level, improving substrate utilization in cardiomyocytes. These different, but potentially complementary mechanisms, raise the possibility that metabolic modulation with trimetazidine may provide incremental benefit when used in conjunction with contemporary heart failure therapies. Nevertheless, at present, there are no robust clinical trials that evaluate the combination of trimetazidine with SGLT2 inhibitors, ARNIs, and other guideline-directed therapies. Future research should therefore focus on large, adequately powered multicenter randomized trials evaluating the efficacy of trimetazidine in the era of contemporary heart failure management. Particular emphasis should be placed on determining its role in patients receiving optimal guideline-directed medical therapy, identifying patient subgroups most likely to benefit, including those with ischemic heart disease and diabetes, and assessing long-term effects on hospitalization, mortality, quality of life, and ventricular remodeling.

In view of the various non-ischemic causes of heart failure, a more scrupulous subgroup analysis of different causes of heart failure would be required to understand the effects of trimetazidine in these patients. Studies to assess the effects of trimetazidine in HFpEF would also be crucial, as half of the patients with heart failure have a preserved ejection fraction [52]. Additionally, it is also associated with significant morbidity and premature mortality. However, the results of the recent DoPING-HFpEF showed that trimetazidine did not improve myocardial energy homeostasis or exercise hemodynamics in patients with HFpEF [53].

Another recent study in 63 patients with diabetic cardiomyopathy demonstrated improvements in left atrial volume, mitral e’ velocity, and left ventricular GLS after 3 months of trimetazidine therapy [54]. These differing results can be explained by the fact that the DoPING-HFpEF primarily enrolled patients with hypertension without diabetes while the latter enrolled only a diabetic population. A European study is currently investigating the effects of trimetazidine on clinical and biochemical parameters of cardiac function and on overall health outcomes in patients with HFpEF [55].

Instead, the TACT-TTR Study (NCT07836699) is evaluating the role of trimetazidine in transthyretin cardiac amyloidosis.

## 8. Limitations of This Review

This is a narrative rather than a systematic review. Source selection reflected author judgement, no formal risk-of-bias assessment was performed, and no quantitative synthesis was attempted. Publication bias cannot be excluded, particularly given that much of the trial evidence originates from single-center studies with modest sample sizes and from meta-analyses that overlap substantially in their included trials. The effect estimates cited should therefore be interpreted as indicative rather than definitive, and the conclusions are appropriately qualified.

## 9. Conclusions

Data from several studies indicate that congestive heart failure can be treated with trimetazidine as an adjunct therapy, especially in patients with ischemic and non-ischemic cardiomyopathy. Trimetazidine’s capacity to alter fatty acid oxidation towards glucose utilization within the myocardium improves the efficiency of the latter as well as its ability to withstand oxidative damage. Observed benefits include a significant increase in exercise tolerance, an improvement in quality of life, and enhanced left ventricular function. The positive trends regarding reduction in hospitalization rates and all-cause mortality is further evidence that this drug is used in the management of heart failure. Data have also indicated a potential role for trimetazidine in patients with diabetic cardiomyopathy, while studies are currently underway regarding its effects in patients with amyloidosis-related cardiomyopathy and HFpEF.

Further research, specifically concerning HFpEF, is required to fully define the extent of the benefits offered and thereby broaden the scope of its application. Consequently, optimizing the use of trimetazidine in the treatment of chronic heart failure will require the adoption of personalized therapeutic strategies that take into account the underlying cause and any existing comorbidities.

## Figures and Tables

**Table 1:** Randomized controlled trials: study characteristics.

Author, Year	N	Age	Duration	Etiology (%)	NYHA Class	LVEF (%) Pre-Treatment
Belardinelli et al., 2001 [10]	38	50–54	2 months	Ischemic cardiomyopathy	II–III	33
Belardinelli et al., 2007 [11]	51	51–52	1 months	Ischemic cardiomyopathy		33
Belardinelli et al., 2008 [12]	35	54	3 months	Ischemic cardiomyopathy		37–38
Cera et al., 2010 [13]	30	65–70	6 months	Ischemic (60%) and non-ischemic (40%) cardiomyopathy	I–III	36
Di Napoli et al., 2005 [14]	61	67–69	18–48 months	Ischemic and dilated cardiomyopathy		31
Di Napoli et al., 2007 [15]	50	67–69	6 months	Ischemic cardiomyopathy	II–IV	28–29
El-Kady et al., 2005 [16]	200	53	23 months	Ischemic cardiomyopathy		36
Fragasso et al., 2006 [17]	65	64–66	13 months	Ischemic (54%) and non-ischemic (46%) cardiomyopathy	II–IV	35
Fragasso et al., 2011 [18]	44		36 months	Ischemic (66%) and non-ischemic (34%) cardiomyopathy		<45
Gunes et al., 2009 [19]	87	57–59	3 months	Ischemic (66%) and non-ischemic (29%) cardiomyopathy	II–III	32
Jatain et al., 2016 [20]	100	47.7	3 months	Non-ischemic (46%) cardiomyopathy		27
Momen et al., 2016 [21]	98	58.5	6 months	Ischemic, dilated cardiomyopathy		<40
Rosano et al., 2003 [22]	32	65–66	6 months	Diabetic cardiomyopathy		33
Sisakian et al., 2007 [23]	82	64–66	3 months	Ischemic cardiomyopathy,	II–III	32–34
Thrainsdottir et al., 2004 [24]	20	66–67	4 weeks	Ischemic (50%) and Non-ischemic (50%) cardiomyopathy	II–III	<40%
Tuunanen et al., 2008 [25]	19	57–59	3 months	Idiopathic Dilated Cardiomyopathy	II	34
Vitale et al., 2004 [26]	47	77–78	6 months	Ischemic cardiomyopathy	I–III	28–29
Yuanhong, et al., 2017 [27]	60	NA		Left bundle branch block (LBBB) patients with dilated cardiomyopathy		38
Zemljic et al., 2010 [28]	47	65–66	1 months	Ischemic cardiomyopathy	II–III	<55

NYHA: New York Heart Association; LVEF: left ventricular ejection fraction.

**Table 2:** Change in NYHA functional class in patients treated with and without trimetazidine along with conventional therapy.

		Class I	Class II	Class III	Class IV	Improvement/Deterioration
Fragasso 2006 [17]	TMZ	↑ 11%	↑ 53%	↓ from 75% to 11%	-	Decreased by: 1 grade in 21 patients (75%) 2 grades in 1 patient (3.57%)
	CT			↑ from 48% to 67%	↑ from 4% to 11%	Increased by 1 grade in 9 patients (33.33%) 2 grades in 2 patients (7.40%)
Jatain 2016 [20]	TMZ	-	-	-	-	Improved by 1–2 grades in 37% patients, 0% deterioration
	CT	-	-	-	-	Deteriorated by grade 1–2 in 11.5% patients
Vitale 2004 [26]	TMZ	↑ 17.93%	↓ 13.04%	↓ 8.69%	0%	Improved by 1–2 grades in 39.66% patients
	CT	↓ 4.5%	↓ 20.84%	↑ 4.17%	↑ 12.5%	Deteriorated by 1–2 grades in 16.67% patients

TMZ: Trimetazidine; CT: Conventional therapy; NYHA: New York Heart Association functional class; ↑ increase; ↓ decrease; Hyphen (-) indicates data not reported.

**Table 3:** Effect of trimetazidine on biomarkers of heart failure.

Study	Marker	Effect of Trimetazidine
Di Napoli et al., 2007 [15]	BNP	Significantly reduced from 252 ± 44 pg/mL to 135 ± 22 pg/mL in 6 months; *p* < 0.001 in trimetazidine, whereas it significantly increased in controls from 239 ± 59 pg/mL to 288 ± 46 pg/mL; *p* < 0.02.
Di Napoli et al., 2007 [15]	cTnT	Significantly reduced from 0.7 pg/mL to 0.1 pg/mL in trimetazidine, while it remained unchanged (0.8 pg/mL) in the control group.
Fragasso et al., 2006 [17]	BNP	Significantly decreased in patients on trimetazidine from 683 ± 362 pg/mL to 349 ± 211 pg/mL; *p* < 0.01. Remained unchanged in the conventional therapy group (from 575 ± 416 pg/mL to 522 ± 291 pg/mL, *p* = 0.2).
Jatain et al., 2016 [20]	BNP	Significant fall from 744.7 ± 834.9 pg/mL to 248.3 ± 278.5 pg/mL, −56.8% reduction at 3 months while patients on conventional therapy exhibited 25.6% reduction from 712.9 ± 606.8 pg/mL to 455.44 ± 475.87 pg/mL.
Yuanhong et al., 2017 [27]	NT-pro BNP	Significantly decreased in the trimetazidine group from 3.11 ± 0.47 pg/mL to 2.77 ± 0.45 pg/mL, *p* < 0.01.

BNP: brain natriuretic peptide; NT-pro-BNP: N-terminal pro-brain natriuretic peptide.

**Table 4:** Characteristics of meta-analyses evaluating trimetazidine in patients with ischemic and non-ischemic heart failure.

Author, Year	No. of Studies	n (Total)		Heart Failure Population	Study Objective
		TMZ	Control		
Ajabnoor 2022 [37]	6	171	167	IHD patients not suitable for revascularization	To investigate the effect on functional and exercise measurements when compared to first-line antianginal drugs alone.
Zhao 2021 [38]	06	161	149	Non-ischemic	To determine the efficacy of TMZ versus conventional therapy.
Grajek 2015 [35]	3	164	162	Ischemic	To verify whether the marked improvement provided by TMZ in echocardiographic and clinical parameters was likely to translate into reduced ACM in systolic HF patients.
Zhou 2014 [34]	19	537	457	Ischemic and non-ischemic	To provide more convincing evidence of TMZ therapy in patients with CHF.
Zhang 2012 [39]	16	455	429	Ischemic and non-ischemic	To evaluate the effects of additional TMZ treatment on patients with CHF.
Gao 2011 [40]	17	955		Ischemic and non-ischemic	To explore whether trimetazidine could improve symptoms, cardiac functions, and clinical outcomes in patients with heart failure.

ACM: All-Cause Mortality; TMZ: Trimetazidine; CHF: Chronic Heart Failure; IHD: Ischemic Heart Disease.

**Table 5:** Meta-analyses of studies in patients with both ischemic and non-ischemic heart failure: trimetazidine + conventional therapy vs. conventional therapy alone.

Parameter	Zhang 2011 [39]	Gao 2011 [40]	Zhaou 2014 [34]	Nassiri 2024 [41]
LVEF % (WMD)	6.46%; *p* < 0.0001	Ischemic: 7.37%; *p* < 0.01 Non-Ischemic: 8.72%; *p* < 0.01	7.29%; *p* < 0.01	-
TED (s) (WMD)	63.75; *p* < 0.0001	30.26; *p* < 0.01	No significant differences	-
NYHA Class (WMD)	−0.57; *p* < 0.0003	−0.41; *p* < 0.01	−0.55; *p* < 0.01	−0.44; 95% CI: −0.49–0.39
LVESD (mm) (WMD)	−6.67; *p* < 0.0001	-	-	-
LVEDD (mm)	−6.05; *p* < 0.0001	-	-	-
LVESV (mL) (WMD)	-	−10.37; *p* < 0.01	−17.09; *p* < 0.01	-
LVEDV (mL) (WMD)	-	-	11.24; *p* < 0.01	-
Hospitalization (RR)	-	0.42; *p* < 0.00001	0.43; *p* = 0.03	0.42; 95% CI: 0.29–0.60
ACM (RR)		0.29; *p* < 0.00001	No significant differences	-
BNP (pg/mL) (WMD)	−203.40; *p* = 0.0002	-	−157.08; *p* < 0.01	-

BNP: Brain Natriuretic Peptide; CI: Confidence Interval; LVEF: Left Ventricular Ejection Fraction; LVESD: Left Ventricular End-Systolic Diameter; LVESV: Left Ventricular End-Systolic Volume; LVEDV: Left Ventricular End-Diastolic Volume; NYHA: New York Heart Association; LVEDD: Left Ventricular End-Diastolic Diameter; WMD: Weighted Mean Differences.; TED: Total Exercise Duration. *p* values represent comparisons between trimetazidine plus conventional therapy and conventional therapy alone.

**Table 6:** Meta-analyses of studies in patients with either ischemic and non-ischemic heart failure: trimetazidine + conventional therapy vs. conventional therapy alone.

Parameter	Garjek 2015 [35]	Ajabnoor 2022 [37]	Zhao 2021 [38]
Heart Failure type	Ischemic	Ischemic, not suitable for revascularization	Non-Ischemic
LVEF % (WMD)		1.22; *p* = 0.06	3.09%; *p* = 0.002 6.09%; *p* < 0.0001
6-MWD (m) (WMD)		1.75; *p* < 0.001	48.51; *p* < 0.0001
NYHA Class (WMD)		−1.30; *p* < 0.01	
LVESV (mL) (WMD)			–11.23; *p* = 0.0006
LVEDV (mL) (WMD)			–6.92; *p* = 0.07
ACM (RR)	0.283; *p* < 0.0001		

ACM: All-Cause Mortality; LVEF: Left Ventricular Ejection Fraction; LVESV: Left Ventricular End-Systolic Volume; LVEDV: Left Ventricular End-Diastolic Volume; NYHA: New York Heart Association; 6-WMD: 6-Minute Walking Distance; WMD: Weighted Mean Differences.

## Data Availability

No new data were created or analyzed in this study. Data sharing is not applicable to this article.
